# Retraction: Autophagy in Muscle of Glucose-Infusion Hyperglycemia Rats and Streptozotocin-Induced Hyperglycemia Rats via Selective Activation of m-TOR or FoxO3

**DOI:** 10.1371/journal.pone.0223144

**Published:** 2019-09-23

**Authors:** The PLOS ONE Editors

After publication of this article [1], concerns were raised about similarities between the Actin blots in Figures 4A, B and the p-S6 blot in Figure 4D. The authors explained that errors were made in preparing these figures such that the same data were included in these three panels; they provided raw data and an updated figure.

During our assessment of this matter, members of *PLOS ONE*’s Editorial Board and an external reviewer advised that while the files provided adequately addressed the above concerns about Figure 4, there are several additional concerns that call into question the overall scientific validity of the results reported in the article:

- The experimental design was not adequate for assessment of autophagy or autophagic flux, and hence the article’s conclusions regarding enhancement and inhibition of autophagy are not supported by the data reported. The authors commented that the assessment of autophagy and autophagic flux in vivo has not been well established, and that the detection of autophagy core protein expression, such as LC-3I/II, atg5, atg7 and Beclin1 et al. is an accepted approach, but the consulted experts advised that additional measures were needed.
- Some of the results rely on quantification of non-fluorescent western blots, and furthermore many blots are overexposed.
- Total protein level controls are not provided for phosphoprotein western blot experiments in Figure 4.
- Concerns were raised about reported LC3-I and LC3-II levels reported in Figures 1C, 3A, and 3B. Concerns were also raised about differences in CON blot data reported in Figures 4A, B as compared to CON data in Figures 4C, D, and that the vehicle controls may have driven the overall conclusions of these experiments. The authors noted that the LC3 levels of the different animal models or control groups were not comparable, animals were treated differently and so differed in physiological parameters such as blood volume and components.
- The western blot figures report data from three experimental replicates although the Methods specify that the experiments included six replicates. The authors clarified that only three samples per experimental group were reported for the western blot analyses, but the full experiments investigating metabolic profiles included six rats per group.

The *PLOS ONE* Editors considered the authors’ comments and the input received from our external advisors. Per our assessment, the concerns about the study design and controls have not been adequately addressed, and so we retract this article due to concerns about the validity and reliability of the reported results. We regret that these issues were not adequately addressed during pre-publication peer review.

The authors did not agree with the retraction.
